# Supplementary material for: Plasmonic layer-selective all-optical switching of magnetization with nanometer resolution
Source: Nat Commun. 2019 Oct 21;10:4786. doi: 10.1038/s41467-019-12699-0 (PMC6803660; doi:10.1038/s41467-019-12699-0)
Supplement: Supplementary file 1 — Supplementary Information [file 41467_2019_12699_MOESM1_ESM.pdf]

Plasmonic layer-selective all-optical switching of magnetization with nanometer resolution

D. O. Ignatyeva,[1,2,*,a] C. S. Davies,[3,4,*,b] D. A. Sylgacheva,[1,2] A. Tsukamoto,[5] H. Yoshikawa,[5] P. O. Kapralov,[2] A. Kirilyuk,[4] V. I. Belotelov[1,2] and A. V. Kimel[3,6]

[1] *Faculty of Physics, Lomonosov Moscow State University, 119991, Moscow, Russia*

[2] *Russian Quantum Center, 45 Skolkovskoye Shosse, 121353, Moscow, Russia*

[3] *Radboud University, Institute for Molecules and Materials, 135 Heyendaalseweg, 6525 AJ Nijmegen, The Netherlands*

[4] *Radboud University, FELIX Laboratory, 7c Toernooiveld, 6525 ED Nijmegen, The Netherlands*

[5] *College of Science and Technology, Nihon University, 7-24-1 Funabashi, Chiba 274-8501, Japan*

[6] *Moscow Technological University (MIREA), 119454, Moscow, Russia*

[*] *D. O. Ignatyeva and C. S. Davies contributed equally to this work*

[a] daria.ignatyeva@gmail.com, [b] c.davies@science.ru.nl

Supplementary material

Supplementary Note 1

In Supp. Fig. 1, we show a schematic of the experimental setup used to achieve and detect layer-resolved all-optical switching. A full description of the setup is given in the Methods. By translating the mirror and lens along the dashed axis, we were able to tune the angle of incidence θ of the optical pulse on the top GdFeCo layer.

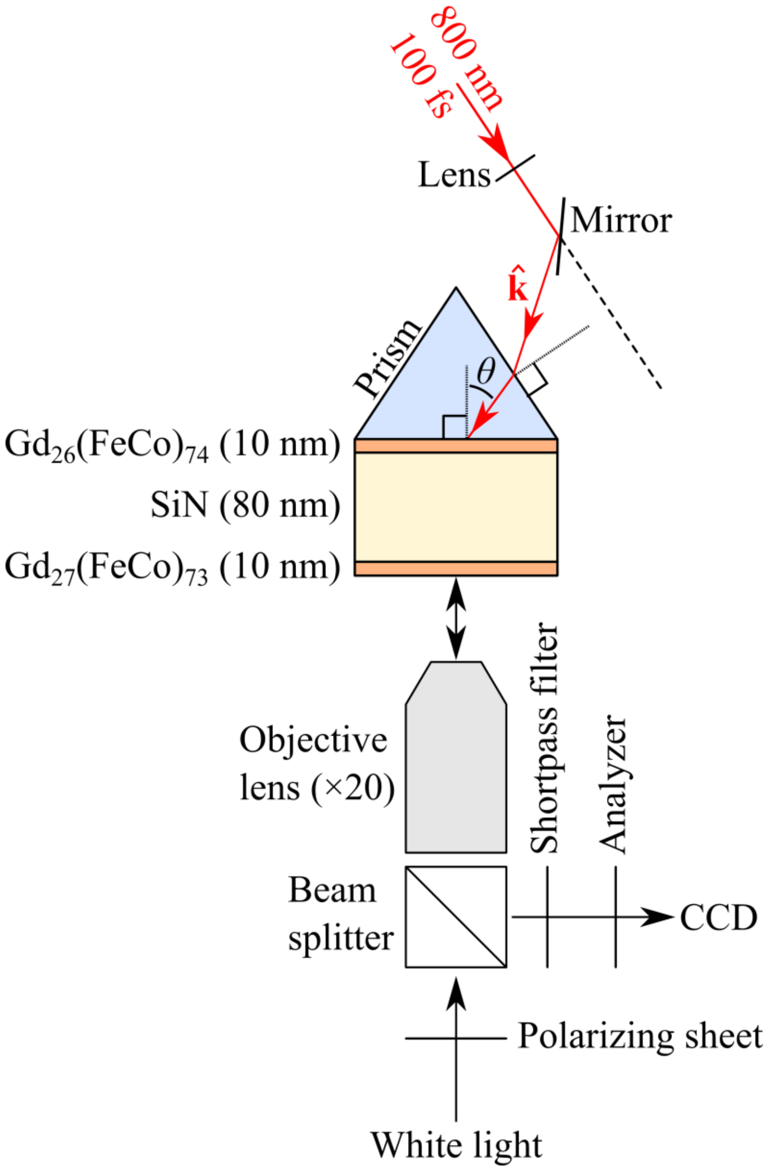

Supp. Fig. 1 Schematic of the experimental setup. Imaging of the magnetization in both layers of GdFeCo was performed using the magneto-optical Kerr effect, with white polarized light serving as the illumination. The optical pulse responsible for magnetization switching was directed through the coupling prism, with its polarization tuned by rotating a half-wave plate.

Supplementary Note 2

In Fig. 3 in the main text, we used cross-sections extracted from background-corrected magneto-optical images to demonstrate that p-polarized and s-polarized optical pulses can switch magnetization independently in different layers. In Supp. Fig. 2, we provide for completeness the cross-sections extracted from the raw magneto-optical images.

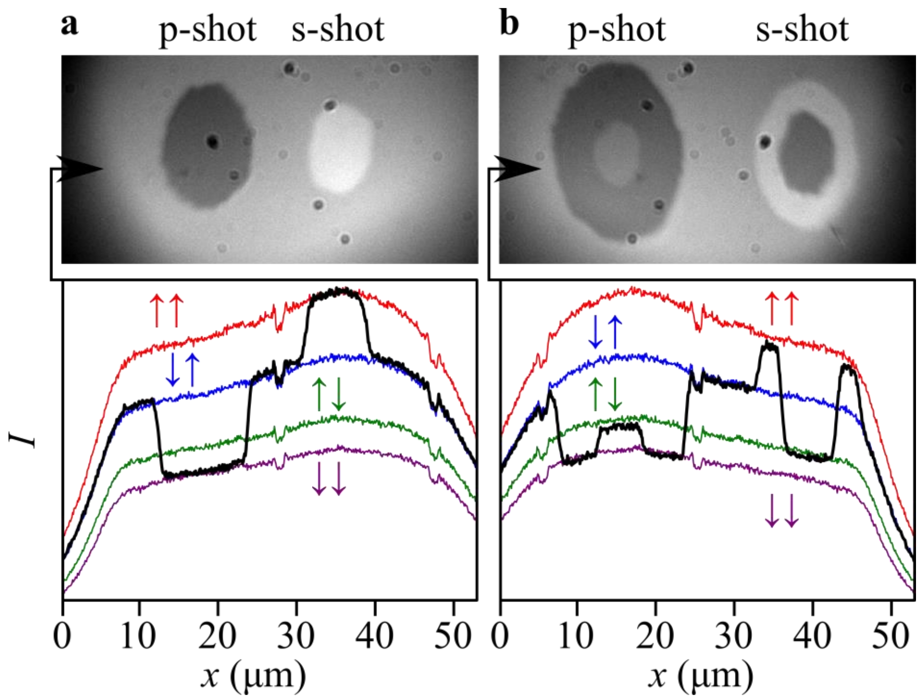

Supp. Fig. 2 Polarization-dependent all-optical switching of magnetization in different layers of the heterostructure. Raw magneto-optical images are shown, taken after exposing the heterostructure to a single p-polarized (left spot) and s-polarized (right spot) optical pulse, incident at an angle of 59°. Also shown is a cross-section (averaged over a width of 1.8 μm) extracted from the image. The left (right) arrow indicates the orientation of the magnetization of Gd within the top (bottom) layer of GdFeCo. The results shown in **a** and **b** were obtained with an incident fluence of ~10 mJ/cm^2 and ~12 mJ/cm^2 respectively.

Supplementary Note 3

In Fig. 3a in the main text, we showed that the polarization-dependent all-optical switching effect can be successfully achieved when the magnetization initially has positive and negative projection on to the sample normal in the top $Gd_{26}(FeCo)_{74}$ and bottom $Gd_{27}(FeCo)_{73}$ layers respectively. In Supp. Fig. 3, we show that the plasmon-enabled magnetization reversal effect can be achieved for all 4 ground magnetic states.

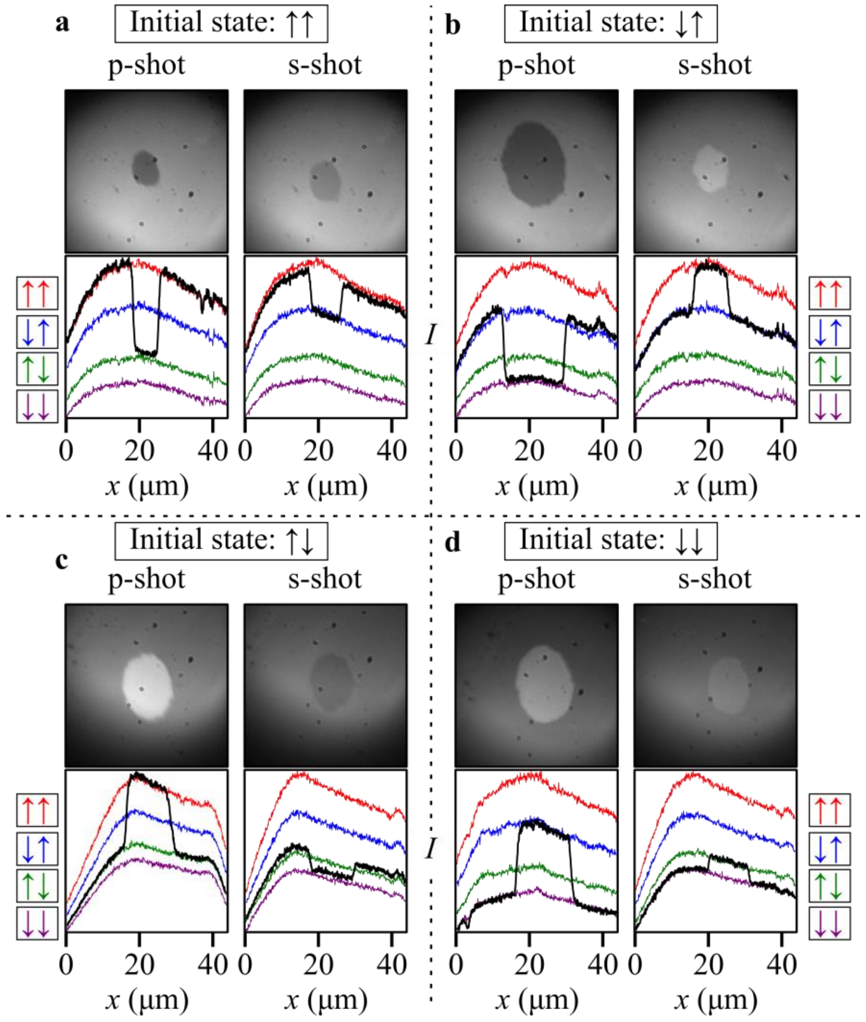

Supp. Fig. 3 Polarization-dependent all-optical switching of magnetization for different intial magnetic states of the heterostructure. **a-d** Raw magneto-optical images are shown, taken after exposing the heterostructure to a single p-polarized (left spot) and s-polarized (right spot) optical pulse, incident at an angle of 59° and with incident fluence between 9 mJ / cm^2 and 10 mJ / cm^2. Also shown is a cross-section (averaged over a width of 0.9 μm) extracted from the image. The left (right) arrow indicates the orientation of the magnetization of Gd within the top (bottom) layer of GdFeCo. The results shown in **a-d** were obtained with different initial magnetic states as indicated.

Supplementary Note 4

In the main text, polarization-dependent all-optical switching was identified using a single ultrashort pulse. In Supp. Fig. 4, we show the effect of exposing the multi-layered heterostructure to multiple consecutive pulses. It is clearly apparent that the magnetization in the targeted layer is reversed after each and every shot (provided that there is no jitter in the pulse energy or spatial position).

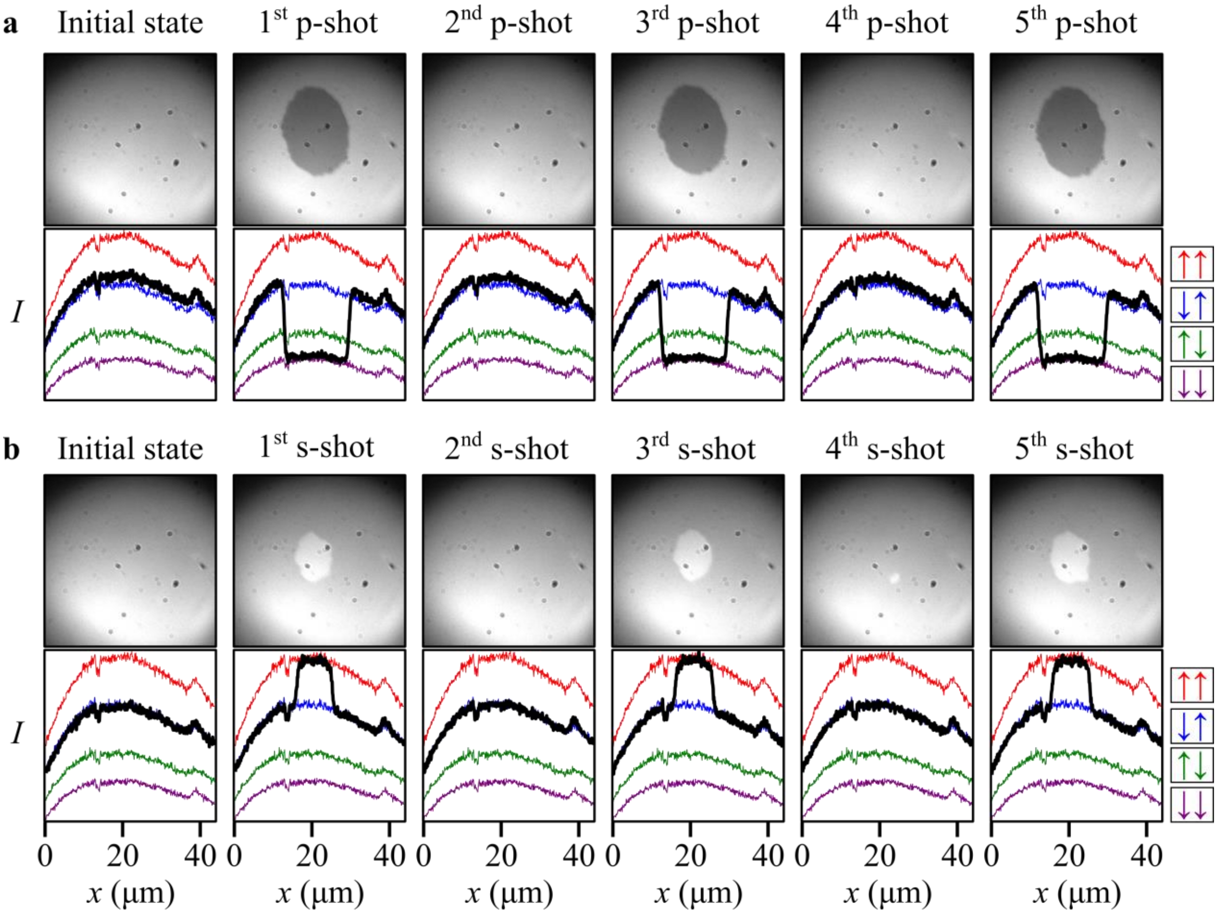

Supp. Fig. 4 Effect of exposing the multi-layered heterostructure to consecutive optical pulses with different polarization. **a-b** Raw magneto-optical images taken after exposing the system to consecutive optical pulses as indicated, at an angle of incidence of 59°. The contrast is linearly proportional to the out-of-plane component of magnetization M_z mainly of the Fe sublattice. Also shown are corresponding cross-sections (averaged over 0.9 μm) extracted from the image (black curve). The other curves correspond to the same cross-section extracted from images of the other spatially-homogenous magnetic states, labelled such that the first and second arrows indicate the magnetization orientation of gadolinium in the top $Gd_{26}(FeCo)_{74}$ and bottom $Gd_{27}(FeCo)_{73}$ layers respectively. The results shown in panels **a** and **b** were obtained with p-polarized and s-polarized optical pulses respectively, with an incident fluence of ~10 mJ / cm^2.

Supplementary Note 5

In the main text, we discussed how varying the angle of incidence θ of the optical pulse influenced whether layer-resolved switching could be achieved. In Supp. Fig. 5, we show the raw magneto-optical images, and corresponding cross-sections, on which this discussion was based. Several points must be clarified about this data set. Firstly, changing the angle of incidence of the 800 nm optical pules required changing of the optical path. We therefore attribute spatial non-uniformities of the switched magnetization (seen in Supp. Fig. 5a,d,f-g) to scattering of the pulse from defects within the glass prism. Secondly, for the case of the p-polarized pulse with angle of incidence of 40° (Supp. Fig. 5f), the incident optical fluence was excessive, resulting in a multi-domain pattern of switched magnetization. We extract the cross-section from the topmost edge of the switched domain, thus showing the p-polarized pulse switches the top $Gd_{26}(FeCo)_{74}$ layer at low optical fluence.

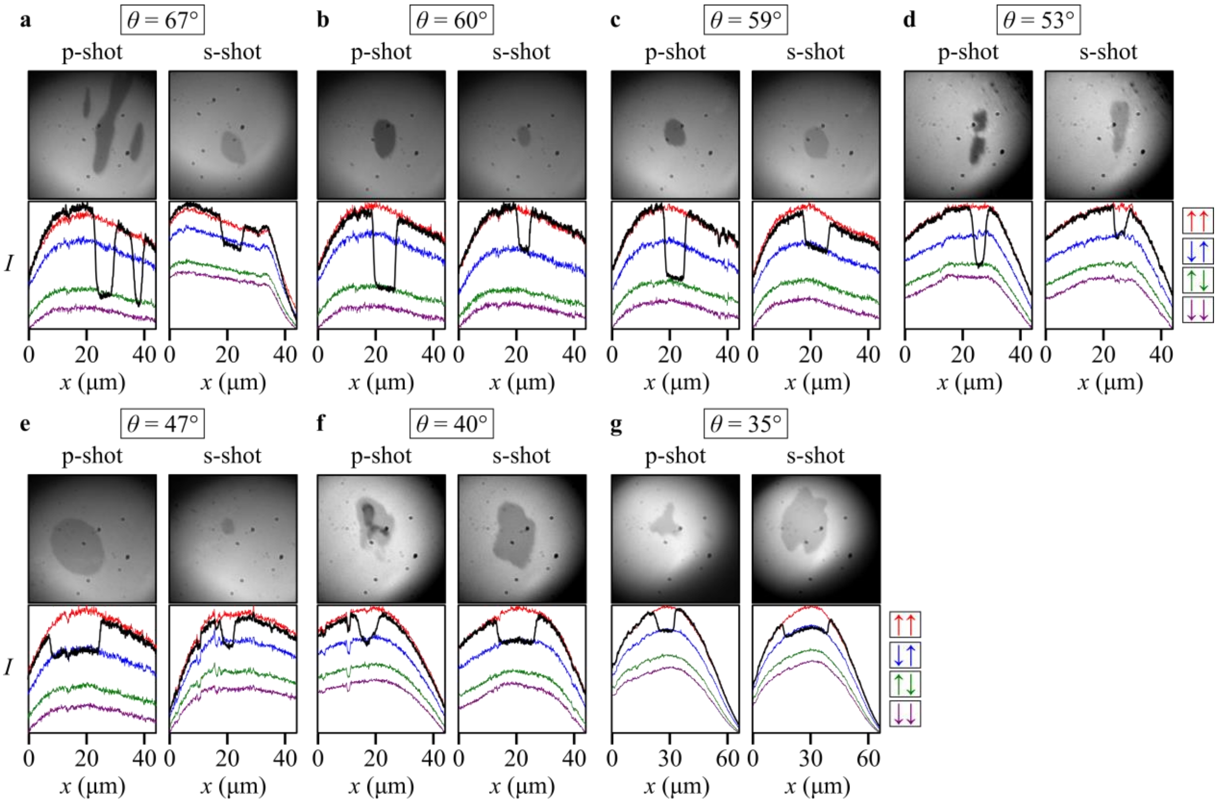

Supp. Fig. 5 Polarization-dependent all-optical switching using an optical pulse with a varying angle of incidence. **a-g** Raw magneto-optical image taken after exposing the heterostructure to a single p-polarized and s-polarized optical pulse. The contrast is linearly proportional to the out-of-plane component of magnetization M_z mainly of the Fe sublattice. Also shown are cross-sections (averaged over 0.9 μm) extracted from the image (black curve). The other curves correspond to the same cross-section extracted from images of the other spatially-homogenous magnetic states, labelled such that the first and second arrows indicate the magnetization orientation of gadolinium in the top $Gd_{26}(FeCo)_{74}$ and bottom $Gd_{27}(FeCo)_{73}$ layers respectively. The results shown in panels **a-g** were obtained with different angles of incidence as indicated.
